# Genomic characterization of *Salmonella* isolates from food and diarrheal patients in Ruian, China

**DOI:** 10.3389/fmicb.2026.1840296

**Published:** 2026-04-29

**Authors:** Na Li, Tao Lv, Bangyao Zhou, Dongying Ni, Huanhuan Ye, Chenchen Qu, Danhua Zhu, Yunbo Chen

**Affiliations:** 1 Ruian Center for Disease Control and Prevention (Ruian Health Supervision Institute), Ruian, China; 2 State Key Laboratory for Diagnosis and Treatment of Infectious Diseases, The First Affiliated Hospital, Zhejiang University School of Medicine, Hangzhou, China; 3 Jinan Microecological Biomedicine Shandong Laboratory, Jinan, China

**Keywords:** antimicrobial resistance, diarrheal patient, food, *Salmonella*, serotype distribution, virulence, whole-genome sequencing

## Abstract

**Introduction:**

Salmonella is a major foodborne pathogen threatening public health, and the cross-transmission between food and human sources remains a critical concern. To elucidate the epidemiological characteristics and potential transmission of *Salmonella* in Ruian City, China, we conducted a comprehensive phenotypic and genomic analysis of 173 archived *Salmonella* isolates collected from patients (*n* = 101) and food samples (*n* = 72) during 2020–2021.

**Methods:**

We serotyped the strains and determined their antimicrobial resistance phenotypes, and used whole-genome sequencing(WGS) to analyze virulence genes, resistance genes, and the genetic relatedness among the isolates.

**Results:**

A total of 28 distinct serovars were identified, with *S*. Typhimurium being the most prevalent in clinical isolates (26.73%) and *S.* London the dominant serovar in food isolates (29.85%); *S*. Typhimurium, *S.* I 4,[5],12:i:-, *S.* London, *S.* Rissen and *S.* Goldcoast were detected in both sources. Clinical and food isolates exhibited high resistance rates to amoxicillin (72.28%/47.22%), piperacillin (67.33%/43.06%), minocycline (42.57%/41.67%), and trimethoprim-sulfamethoxazole (34.65%/40.28%), with multidrug-resistant (MDR) rates of 43.56 and 31.94%, respectively. Statistically significant differences in resistance to amoxicillin, piperacillin, gentamicin and minocycline were observed between *S*. Typhimurium and *S.* London. WGS identified 31 STs, with high concordance between serovar and ST. A total of 91 antimicrobial resistant genes (ARGs) were detected, with *aac(6′)-Iaa* (67.05%) being the most prevalent aminoglycoside resistance gene and *bla*_TEM-1_ being the dominant β-lactamase gene; a discrepancy between ARGs and phenotype was observed for several antibiotics. *S.* Rissen harbored fewer virulence genes (e.g., lacking *lpfD*, *ratB*, *sodC1*, *sseI* and *sspH2*) and exhibited a gene-content pattern consistent with lower virulence potential. Phylogenomic analysis based on cgSNP showed that isolates of the same serovar clustered together, and cgSNP-based minimum spanning tree revealed close genetic relatedness (SNP ≤ 20) between pork and clinical isolates of *S*. Typhimurium, *S.* London, *S.* Goldcoast and *S.* Rissen, indicating potential cross-transmission between food and human sources.

**Discussion:**

Our findings highlight the dis-tinct serotype distribution, ARGs and virulence characteristics of Salmonella from different sources in Ruian. These data provide a scientific basis for the prevention, control and clinical treatment of salmonellosis, and emphasize the necessity of continuous surveillance and the implementation of the One Health approach to mitigate *Salmonella* transmission along the food chain.

## Introduction

*Salmonella* is a gram-negative genus in the family Enterobacteriaceae that comprises numerous serovars responsible for a wide spectrum of human and animal disease. More than 2,600 serovars of *Salmonella enterica* have been described based on somatic, flagellar and capsular antigens, and these serovars differ markedly in host range, pathogenic potential and epidemiology (Dos Santos et al., 2019). The global food supply and intensive animal production have facilitated the dissemination and host-range expansion of many serovars via horizontal gene transfer and long-distance distribution of contaminated products (Fang et al., 2022). Foodstuffs—especially poultry, pork, eggs, beef and some vegetables—are frequent vehicles of *Salmonella* transmission to humans, with pork and chicken recognized as major reservoirs in many settings (Baek et al., 2024; Ehuwa et al., 2021; Soliani et al., 2023).

The clinical manifestations of *Salmonella* infection are heterogeneous. Typhoidal and paratyphoidal serovars (e.g., *S.* Typhi and *S.* Paratyphi A, *S.* Paratyphi B, *S.* Paratyphi C, and *S.* Sendai) typically produce systemic, invasive illness characterized by sustained high fever and extra-intestinal involvement, whereas non-typhoidal *Salmonella* (NTS) most often causes self-limited gastroenteritis with diarrhea, abdominal cramps and fever (Cheng et al., 2019). Nevertheless, certain populations—including infants, elderly persons and immunocompromised patients—are at elevated risk of severe or invasive outcomes such as bacteremia, meningitis, endovascular infection and osteomyelitis (Lamichhane et al., 2024). Globally, *Salmonella* remains a major cause of diarrheal disease and death, and in China it accounts for a substantial proportion of foodborne bacterial outbreaks and a high estimated incidence of NTS (Besser, 2018; Dong et al., 2015; Liu et al., 2021; Xu Y. et al., 2020).

Antimicrobial resistance (AMR) in *Salmonella* has emerged as a critical public-health concern. Extensive antibiotic use in animal husbandry for therapy and growth promotion has accelerated the selection and dissemination of resistant and multidrug-resistant (MDR) strains, which may traverse the food chain and compromise clinical treatment options (Barza, 2002; Lai et al., 2014; Wang et al., 2022). Surveillance of phenotypic resistance together with molecular characterization of resistance determinants is therefore essential to guide both veterinary and clinical stewardship and to inform risk mitigation along the farm-to-fork continuum.

Whole-genome sequencing (WGS) provides high-resolution tools for modern *Salmonella* surveillance, enabling accurate serovar inference, detection of ARGs and virulence genes, and source attribution through MLST, cgMLST and SNP-level analyses (Deng et al., 2025; Wu and Hulme, 2021; Yan et al., 2023). However, many prior studies in China have been limited to single serovars or single sources (animal, food or clinical) (Chen et al., 2022; Tang et al., 2022; Yang et al., 2019) and relatively few have integrated comparative phenotypic and WGS analyses across food and human isolates from a One Health perspective. Addressing this gap is important for clarifying transmission pathways and for designing targeted interventions.

In this study, we combined phenotypic testing and WGS to characterize 173 archived *Salmonella* isolates recovered from diarrheal patients and food samples in Ruian, China (2020–2021). Our objectives were to: (a) determine serovar distribution and antimicrobial susceptibility across sources; (b) identify molecular characteristics including sequence types, ARGs and virulence genes and assess genotype–phenotype concordance; and (c) evaluate genetic relatedness and potential cross-transmission between food and human isolates to inform surveillance and control strategies.

## Methods and materials

### Collection and serovar identification of *Salmonella* isolates

A total of 173 archived *Salmonella* isolates collected from Ruian city, Zhejiang Province, China, during 2020–2021 were analyzed in this study. Clinical strains were isolated from patients at a designated hospital. Food samples were obtained from National Food Safety Risk Monitoring Program. Only one isolate was included per patient/sample. Conventional serotyping was performed by slide agglutination with somatic (O) and flagellar (H) antisera from the Statens Serum Institute (SSI, Copenhagen, Denmark) according to the White–Kauffmann–Le Minor scheme (Grimont and Weill, 2007). In silico serovar prediction based on whole-genome sequencing (WGS) data was conducted using the *Salmonella* In Silico Typing Resource (SISTR) for confirmation and comparison with conventional results (Yoshida et al., 2016).

### Antimicrobial susceptibility testing

Antimicrobial susceptibility was determined by broth microdilution according to CLSI (Clinical and Laboratory Standards Institute) (2024) guidelines. A panel of 24 antimicrobial agents representing 12 antimicrobial classes was tested: penicillins (amoxicillin, AMX; piperacillin, PIP); penicillins/β-lactamase inhibitors (amoxicillin-clavulanic acid, AMC; piperacillin-tazobactam, TZP); cephalosporins/β-lactamase inhibitors (cefoperazone/sulbactam, CSL; ceftazidime/avibactam, CZA); cephalosporins (cefazolin, CZO; cefuroxime, CXM; ceftazidime, CAZ; ceftriaxone, CRO; cefepime, FEP); cephamycins/oxacephems (cefoxitin, FOX; moxalactam, MOX); fluoroquinolones (ciprofloxacin, CIP; levofloxacin, LVX); carbapenems (imipenem, IPM; meropenem, MEM; ertapenem, EPM); folate pathway inhibitors (trimethoprim-sulfamethoxazole, SXT); aminoglycosides (amikacin, AMK; gentamicin, GEN); fosfomycins (fosfomycin, FOS); monobactams (aztreonam, ATM); and tetracyclines (minocycline, MIN). *Escherichia coli* ATCC 25922 was used as the quality control strain. Breakpoints were interpreted according to CLSI criteria; isolates non-susceptible to at least one agent in three or more antimicrobial classes were defined as MDR isolate (Magiorakos et al., 2012).

### Whole-genome sequencing and data analysis

#### DNA extraction, library preparation and sequencing

Genomic DNA was extracted from overnight cultures using a commercial kit (Qiagen, Beijing, China) following the manufacturer’s instructions. DNA purity and concentration were assessed using a Qubit 4.0 fluorometer (Thermo Fisher Scientific, USA) and 1% agarose gel electrophoresis. Sequencing libraries were prepared using the Nextera XT DNA Library Preparation Kit (Illumina, San Diego, CA, USA) and sequenced on an Illumina HiSeq 2500 platform (paired-end 150 bp).

#### Read quality control and *de novo* assembly

Raw sequencing reads were processed using fastp (v0.23.2) to trim adapters and remove low-quality bases (*Q* < 20). High-quality reads were assembled de novo using SPAdes (v3.13.0) with the “--careful” flag enabled. The quality of the assemblies was evaluated using QUAST (v5.2.0); assemblies showing high contamination or low N50 values (<30,000 bp) were excluded. Raw reads and assembled genomes were deposited in the NCBI SRA/GenBank under accession number PRJNA1436825.

#### In silico typing and MLST

Sequence types (STs) were assigned using the PubMLST database^1^ by querying the assembled sequences. The scheme is based on the allelic profiles of seven housekeeping genes: *aroC*, *dnaN*, *hemD*, *hisD*, *purE*, *sucA*, and *thrA*.

#### Antimicrobial resistance and virulence profiling

Acquired antimicrobial resistance genes (ARGs) and chromosomal point mutations were identified using ResFinder (v4.1) with a minimum sequence identity threshold of 85% and a minimum coverage of 60%. Phenotypic-genotypic concordance was evaluated by comparing predicted resistance determinants with experimental susceptibility data. Virulence-associated genes were identified by searching against the Virulence Factor Database (VFDB) using ABRicate (v1.0.1) with identity and coverage thresholds set at 85 and 60%, respectively. Presence/absence matrices for key virulence loci were visualized using pheatmap (v1.0.12) in R (version 4.x).

#### Phylogenetic analysis and transmission clustering

Core genome alignment was performed using Snippy (v4.6.0). Recombination events were identified and masked using Gubbins (Croucher et al., 2015). Pairwise cgSNP distances were calculated to determine genetic relatedness. A minimum spanning tree (MST) was constructed based on cgSNP distances using GrapeTree (v1.5.0) to visualize genetic clusters and potential transmission links. Transmission between food and human sources was inferred using a cutoff of ≤20 SNPs, as previously reported (Mao et al., 2025). Allelic profiles were generated via the EnteroBase *Salmonella* scheme. A maximum-likelihood (ML) tree was constructed using RAxML (v8.2.12) under the GTRGAMMA model with 1,000 bootstrap replicates and visualized using iTOL (v6).

#### Statistical analysis

All statistical analyses were performed in R (version 4.x) and SPSS (version 28.0). Categorical variables (serovar distribution, antimicrobial resistance rates, and presence/absence of specific resistance or virulence genes) were compared between groups using the Chi-square test or Fisher’s exact test, as appropriate (based on expected cell counts). For count-type outcomes (e.g., number of resistance genes per isolate), between-group comparisons were performed using the Mann–Whitney *U* test.

#### Ethical statement

The study used archived bacterial isolates and de-identified clinical data; ethical approval was obtained from the Clinical Research Ethics Committee of the First Affiliated Hospital, Zhejiang University School of Medicine (approval no. 0273) and all procedures were performed in accordance with relevant guidelines and regulations.

## Results

### Sample overview and isolate distribution

The 173 archived *Salmonella* isolates originated from two sources: clinical specimens from patients (*n* = 101) and food samples (*n* = 72). Food isolates were obtained from retail raw pork (*n* = 67), chicken meat (*n* = 3), frozen dumplings (*n* = 1) and oysters (*n* = 1). Metadata recorded for each isolate included collection date, source (patient or food and, for food, specific commodity), and specimen location; these details are summarized in Tables 1–3.

**Table 1:** Demographic and clinical characteristics of clinical *Salmonella* isolates.

Characteristic	Category	No. of isolates (*n*)	Proportion (%)
Age group	<1 year	6	5.9%
	1–6 years	16	15.8%
	7–18 years	5	5.0%
	19–64 years	51	50.5%
	≥65 years	21	20.8%
	Unknown	2	2.0%
Sex	Male	63	62.4%
	Female	35	34.7%
	Unknown	3	3.0%
Specimen type	Stool	99	98.0%
	Blood	2	2.0%
Collection year	2020	42	41.6%
	2021	59	58.4%

Clinical isolates totaled 101.

**Table 2:** Temporal distribution of clinical *Salmonella* isolates by month and year.

Year	Month	No. of clinical isolates	Major serovar (no. of isolates)
2020	January	3	Kentucky (1), Typhimurium (1), I 4,[5],12:i:- (1)
2020	March	3	Enteritidis (1), Typhimurium (1), Paratyphi A (1)
2020	June	11	Enteritidis (4), London (2), Typhimurium (3), Kentucky (1), Muenchen (1)
2020	July	11	Enteritidis (1), Typhimurium (6), I 4,[5],12:i:- (1), Kentucky (2), Goldcoast (1)
2020	August	11	Kentucky (2), London (3), Typhimurium (3), Enteritidis (1), Goldcoast (1), Corvallis (1)
2020	September	3	Infantis (1), Weltevreden (1), Typhimurium (1)
2021	January	1	Typhimurium (1)
2021	February	2	Enteritidis (1), Typhi (1)
2021	April	2	London (1), Enteritidis (1)
2021	May	8	Enteritidis (1), I 4,[5],12:i:- (4), Typhimurium (1), London (1), Saintpaul (1)
2021	June	11	London (5), I 4,[5],12:i:- (2), Typhimurium (2), Enteritidis (1), Meleagridis (1)
2021	July	11	I 4,[5],12:i:- (3), Typhimurium (4), Litchfield (1), Rissen (1), Bovismorbificans (1), Goldcoast (1)
2021	August	9	London (3), Typhimurium (2), Enteritidis (2), I 4,[5],12:i:- (2)
2021	September	4	London (1), Enteritidis (2), Kentucky (1)
2021	October	8	Typhimurium (1), Goldcoast (2), Minnesota (1), Enteritidis (1), I 4,[5],12:i:- (1), Oranienburg (1), London (1)
2021	November	3	Enteritidis (1), Typhimurium (1), I 4,[5],12:i:- (1)

A peak in clinical cases was observed during June–August 2020 and May–August 2021, with *S. typhimurium* predominating in 2020, and S. London and I 4,[5],12:i:- increasing in proportion during 2021.

**Table 3:** Distribution of food *Salmonella* isolates by sample type and geographic origin.

Sample type	Collection period	Collection site	No. of isolates (n)	Major serovar (no. of isolates)
Raw pork	Apr-21	AY, YH, DS, SW, TT, FY, NB, XJ, JH, HA, TX	15	London (6), Anatum (2), Rissen (2), Cerro (1), I 4,[5],12:i:- (1), Tennessee (1), Agona (1), Typhimurium (1)
	Jun-21	SC, TT, FY, NB, XJ, TX, CQ, HA, TS, HL, MY, PYK, YZ, TP, PD, LC	25	London (7), Rissen (7), Derby (2), Mbandaka (2), I 4,[5],12:i:- (2), Typhimurium (2), Goldcoast (1), Cerro (1), Senftenberg (1)
	Nov-21	SC, TX, CQ, GL, PYK, AY, SW, DS, TT, NB, TP, MY, YH, FY, TS, XJ	27	London (7), Rissen (7), Manhattan (4), Derby (2), I 4,[5],12:i:- (2), Goldcoast (2), Bovismorbificans (2), Infantis (1)
Chicken meat	May-20	DS, NB, XJ	3	Javiana (1), Infantis (1), Typhimurium (1)
Oysters	Jun-21	MY	1	Derby (1)
Frozen dumplings	Nov-21	JH	1	London (1)

Food and environmental isolates totaled 72. Geographic abbreviations represent subdistricts or townships as indicated in the original data.

### Serotype distribution

Of the 173 tested strains, 172 strains exhibited 100% consistency between their serotype determined using the slide agglutination assay and those predicted in silico from WGS. The only exception was an autoagglutinating strain from a patient, which was assigned to *S.* Typhimurium based on sequencing data. In total, 28 serovars were identified, with 19 in clinical isolates and 16 in food samples (Table 4). To assess source-specific enrichment, we performed 2 × 2 contingency table analyses using Fisher’s exact test (two-sided) for each serovar., followed by Benjamini–Hochberg correction for multiple comparisons (*n* = 28 tests, FDR < 0.05). *S*. Typhimurium was significantly more frequent in clinical isolates than in food [27/101 (26.7%) vs. 4/72 (5.6%); OR = 6.21, 95% CI: 2.10–23.6, adjusted *p* = 2.22 × 10^−4^]. Similarly, *S*. Enteritidis and *S.* Kentucky were detected exclusively in clinical samples and showed significant enrichment (each adjusted *p* < 3.11 × 10^−7^). In contrast, *S.* Rissen was significantly enriched in food sources [1/101 (1.0%) vs. 16/72 (22.2%); OR = 0.04, adjusted *p* = 3.11 × 10^−7^], consistent with its prevalence in pork. *S.* Derby and *S.* Manhattan were also food-exclusive and significantly enriched (each adjusted *p* < 7.62 × 10^−5^). No significant differences were observed for *S.* London [17/101 (16.8%) vs. 21/72 (29.2%); adjusted *p* = 0.40] or *S*. I 4,[5],12:i:- [15/101 (14.9%) vs. 5/72 (6.9%); adjusted *p* = 0.35]. These findings suggest distinct transmission dynamics, with certain serovars bridging clinical and food reservoirs in Ruian during 2020–2021.

**Table 4:** Distribution of serotype and sequence typing (ST) of *Salmonella* isolates.

Serovar	ST	Source		Total (*n*)
		Clinical (*n*)	Food (*n*)	
Typhimurium	ST19/ST1544	27	4	31
I 1,4,[5],12:i:-	ST34	15	5	20
London	ST155	17	21	38
Enteritidis	ST11	17	NA	17
Rissen	ST469	1	16	17
Kentucky	ST198	7	NA	7
Goldcoast	ST2529/ST358	5	3	8
Derby	ST40	NA	5	5
Manhattan	ST18	NA	4	4
Bovismorbificans	ST1499/ST150	1	2	3
Infantis	ST32	1	2	3
Anatum	ST64	NA	2	2
Cerro	ST1593	NA	2	2
Mbandaka	ST413	NA	2	2
Agona	ST13	NA	1	1
Tennessee	ST319	NA	1	1
Senftenberg	ST14	NA	1	1
Javiana	ST9074	NA	1	1
Muenchen	ST82	1	NA	1
Corvallis	ST7010	1	NA	1
Litchfield	ST214	1	NA	1
Weltevreden	ST365	1	NA	1
Oranienburg	ST23	1	NA	1
Saintpaul	ST49	1	NA	1
Meleagridis	ST463	1	NA	1
Minnesota	ST548	1	NA	1
Typhi	ST1	1	NA	1

ST, sequence type; NA, not available.

### Sequence types (MLST) and population structure

Multilocus sequence typing (MLST), based on seven housekeeping loci, effectively discriminated the isolates and showed good concordance with serotyping (Table 4). A total of 31 sequence types (STs) were identified among 171 isolates; two isolates (one *S*. I 4,[5],12:i:- and one *S.* Kentucky) failed to yield valid STs. Most serovars were represented by one or two STs. Specifically, *S.* London corresponded to ST155, *S.* Enteritidis to ST11, *S.* Kentucky to ST198, and *S.* Rissen to ST469. *S*. Typhimurium isolates were predominantly ST19 (*n* = 30) with a single ST1544. All *S*. I 4,[5],12:i:- were ST34 except for one non-typeable isolate. *S.* Goldcoast comprised two STs (ST358 and ST2529): ST2529 was found only in two pork isolates, whereas ST358 occurred in both pork and patient isolates. Among three *S.* Bovismorbificans isolates two STs were observed (ST1499 from a patient and ST150 from pork). The ST distributions are summarized in Table 4, reflecting both host-associated clustering and occasional cross-source sharing of the same STs.

### Antimicrobial susceptibility profiles

A total of 36 isolates were fully susceptible to all antimicrobials tested, including 17 *S.* London, three *S.* Typhimurium, two *S.* Goldcoast, one *S*. Enteritidis and several low-frequency serovars. Resistance rates for each antimicrobial are summarized in Table 5. Clinical isolates showed the highest resistance to amoxicillin (72.3%), piperacillin (67.3%) and minocycline (42.6%), while all clinical isolates remained susceptible to ceftazidime/avibactam, moxalactam and the tested carbapenems. Overall, 43.6% of clinical isolates (44/101) met our definition of MDR. All seven *S.* Kentucky isolates were MDR, with a consistent profile of resistance to amoxicillin, piperacillin, ciprofloxacin, levofloxacin and minocycline. Food isolates also exhibited notable resistance to amoxicillin (47.2%), piperacillin (43.1%), minocycline (41.7%) and trimethoprim-sulfamethoxazole (40.3%), and remained fully susceptible to ceftazidime/avibactam, moxalactam and carbapenems; levofloxacin and amikacin were also active against all food isolates tested. The MDR rate among food isolates was 31.9% (23/72). Nearly all *S.* Rissen (predominantly from pork) were minocycline-resistant, and 56.3% (9/16) of *S.* Rissen isolates were MDR. Comparison of antimicrobial resistance between the two predominant serovars (*S.* Typhimurium, *n* = 31; *S.* London, *n* = 38) was performed using Fisher’s exact test with Benjamini–Hochberg correction for multiple comparisons. *S.* Typhimurium showed significantly higher resistance than *S.* London to amoxicillin, piperacillin and minocycline (all adjusted *p* < 0.05). In contrast, *S*. London exhibited a significantly higher resistance rate to gentamicin (adjusted *p* < 0.05). No other antibiotics showed significant differences after correction for multiple testing.

**Table 5:** Results of antimicrobial susceptibility of *Salmonella* isolates from different sources and serovars.

Antimicrobial agents	Code	Rate of human (%)			Rate of food (%)			Total	Resistant rate (%)	
		S	I	R	S	I	R	R	Typhimurium	London
Penicillins										
Amoxicillin^a^	AMX	27.72	0.00	72.28	52.78	0.00	47.22	61.85%	74.19	42.11
Piperacillin^a^	PIP	29.70	2.97	67.33	54.17	2.78	43.06	57.23%	70.97	36.84
Penicillins + β-lactamase inhibitors										
Amoxicillin-clavulanic acid	AMC	64.36	28.7	6.93	87.50	9.72	2.78	5.20	6.45	2.63
Piperacillin/Tazobactam	TZP	88.12	7.92	3.96	100.0	0.00	0.00	2.31	3.23	0.00
Cephalosporins + β-lactamase inhibitors										
Cefoperazone/Sulbactam	CSL	91.09	6.93	1.98	98.61	0.00	1.39	1.73	0.00	0.00
Ceftazidime/Avibactam	CZA	100.0	0.00	0.00	100.0	0.00	0.00	0.00	0.00	0.00
Cephalosporins										
Cefazolin	CZO	62.38	14.8	22.77	90.28	6.94	2.78	14.45	6.45	2.63
Cefuroxime	CXM	74.26	3.96	21.78	95.83	2.78	1.39	13.29	6.45	2.63
Ceftazidime	CAZ	81.19	0.99	17.82	98.61	0.00	1.39	10.98	6.45	2.63
Ceftriaxone	CRO	79.21	0.00	20.79	97.22	0.00	2.78	13.29	6.45	2.63
Cefepime	FEP	83.17	5.94	10.89	97.22	1.39	1.39	6.94	0.00	2.63
Cephamycins/oxacephems										
Cefoxitin	FOX	95.05	1.98	2.97	100.0	0.00	0.00	1.73	6.45	0.00
Moxalactam	MOX	100.0	0.00	0.00	100.0	0.00	0.00	0.00	0.00	0.00
Quinolones										
Ciprofloxacin	CIP	85.15	4.95	9.90	93.06	5.56	1.39	6.36	0.00	7.89
Levofloxacin	LVX	87.13	3.96	8.91	98.61	1.39	0.00	5.20	0.00	0.00
Carbapenems										
Imipenem	IPM	100.0	0.00	0.00	100.0	0.00	0.00	0.00	0.00	0.00
Meropenem	MEM	100.0	0.00	0.00	100.0	0.00	0.00	0.00	0.00	0.00
Ertapenem	ETP	100.0	0.00	0.00	100.0	0.00	0.00	0.00	0.00	0.00
Folate pathway inhibitors										
Trimethoprim-Sulfamethoxazole	SXT	65.35	0.00	34.65	59.72	0.00	40.28	36.99	35.48	42.11
Aminoglycosides										
Amikacin	AMK	96.04	0.00	3.96	100.0	0.00	0.00	2.31	0.00	0.00
Gentamicin^a^	GEN	82.18	0.00	17.82	87.50	0.00	12.50	15.61	3.23	31.58
Fosfomycins										
Fosfomycin	FOS	99.01	0.99	0.00	100.0	0.00	0.00	0.00	0.00	0.00
Monobactams										
Aztreonam	ATM	82.18	1.98	15.84	97.22	0.00	2.78	10.40	0.00	2.63
Tetracyclines										
Minocycline^a^	MNO	41.58	15.84	42.57	38.89	19.44	41.67	42.20	51.61	7.89

S, susceptible; I, intermediate resistance; R, resistant.

a Indicates statistically significant difference between the two serovars was observed.

### Genotypic antimicrobial resistance and genotype–phenotype concordance

**Figure 1:**
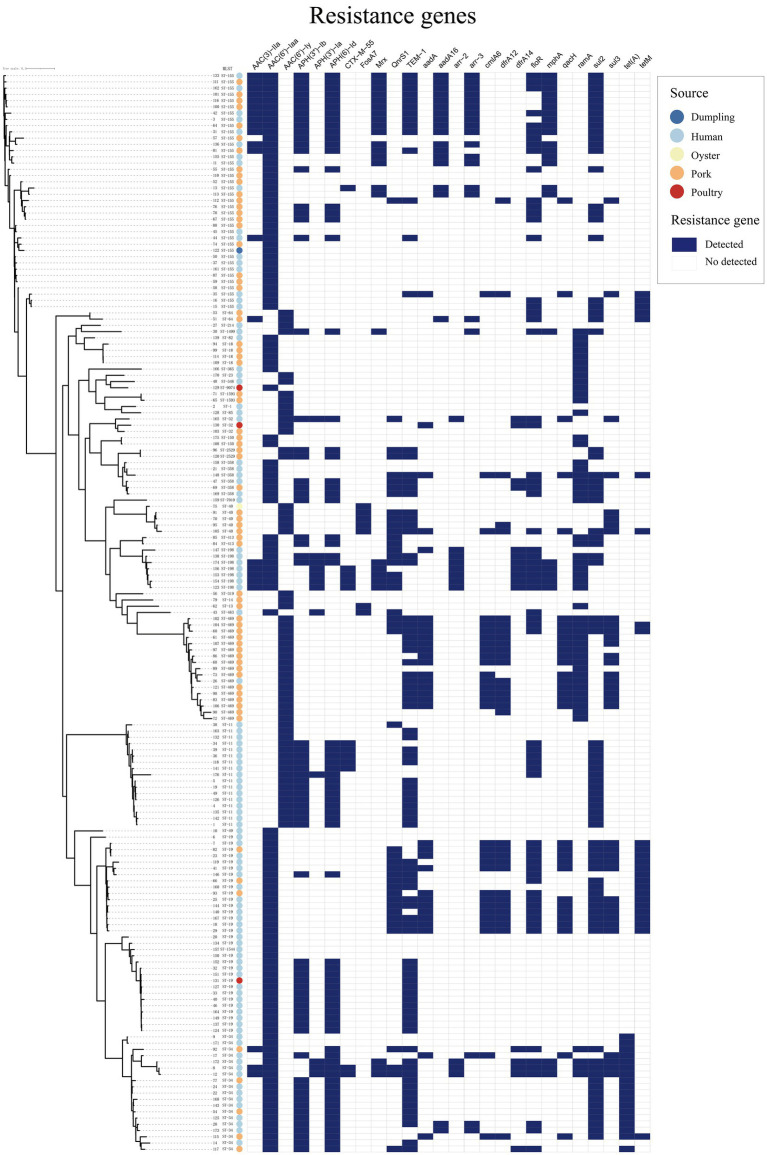
Presence/absence heatmap of antimicrobial resistance genes detected by WGS (*n* = 173). Rows correspond to individual isolates grouped and annotated by serotype and source (clinical vs. food); columns show ARGs ordered by antibiotic class. Dark squares indicate gene detection; light squares indicate absence. A core-genome phylogeny (left) clusters related isolates.

WGS of all isolates identified 91 distinct ARGs. The overall ARGs profile is summarized in Supplementary Table S1 and visualized as a presence/absence heatmap (Figure 1, Supplementary Figure S1). A set of chromosomal/efflux-associated loci (*crp*, *hns*, *acrB*, *acrD*, *bacA*, et al.) was ubiquitous across isolates. Among aminoglycoside-associated genes, *aac(6′)-Iaa* was most common (*n* = 116), followed by *aph(6)-Id* and *aph(3″)-Ib* (each n = 70), *aac(6′)-Iy* (*n* = 57) and *aadA2* (*n* = 33); *rmtB* was limited to four *S.* Kentucky isolates. All gentamicin-resistant isolates carried either *aac(3)-IIa* or *aac(3)-IV* except one. Moreover, strains harboring one of these two genes displayed resistance to gentamicin. This phenomenon indicates a strong correlation between these two resistance genes and the gentamicin resistance phenotype. For β-lactamases resistance genes, *bla*_TEM-1_ was predominant (50.9%), whereas *bla_CTX-M-50_*, *bla_CTX-M-65_*, *bla_TEM-30_*, *bla_TEM-150_*, *bla_OXA-1_*, and *bla_DHA-1_* were only sporadically identified. Overall, 59.5% of the strains were positive for β-lactamases resistance genes. Consequently, the resistant rate to amoxicillin and piperacillin were 61.85 and 57.23%, respectively, showing a strong correlation. Fluoroquinolone-associated *qnrS1* and *qnrB17* were found in 24.9 and 5.8% of isolates, respectively. Among these isolates, only 30.19% were resistant to ciprofloxacin, and 22.64% were resistant to levofloxacin (including intermediate resistance). One isolate exhibited resistance to both ciprofloxacin and levofloxacin; however, no fluoroquinolone-related genes were detected. The overall detection rate of tetracycline resistance genes (*tetA*/*B*/*M*) was 25.29%, significantly lower than the phenotypic resistance rate to minocycline (42.20%) observed in our study. Among strains resistant to trimethoprim-sulfamethoxazole, only 68.8% harbored both *dfr* and *sul* genes. The *floR* efflux gene occurred in 37.6% of strains. Twelve isolates tested were positive for *fosA3* or *fosA7* genes; however, only one of these isolates exhibited intermediate resistance to fosfomycin, while the others remained susceptible. The macrolide resistance gene *mphA* was just observed in 25 strains. Overall, genotypic resistance markers were widespread, but partial discordance with phenotypic susceptibility was observed for some drug classes (notably tetracyclines, fosfomycin and fluoroquinolones), indicating that genotype alone does not fully predict phenotype in this collection.

### Virulence gene profiles

**Figure 2:**
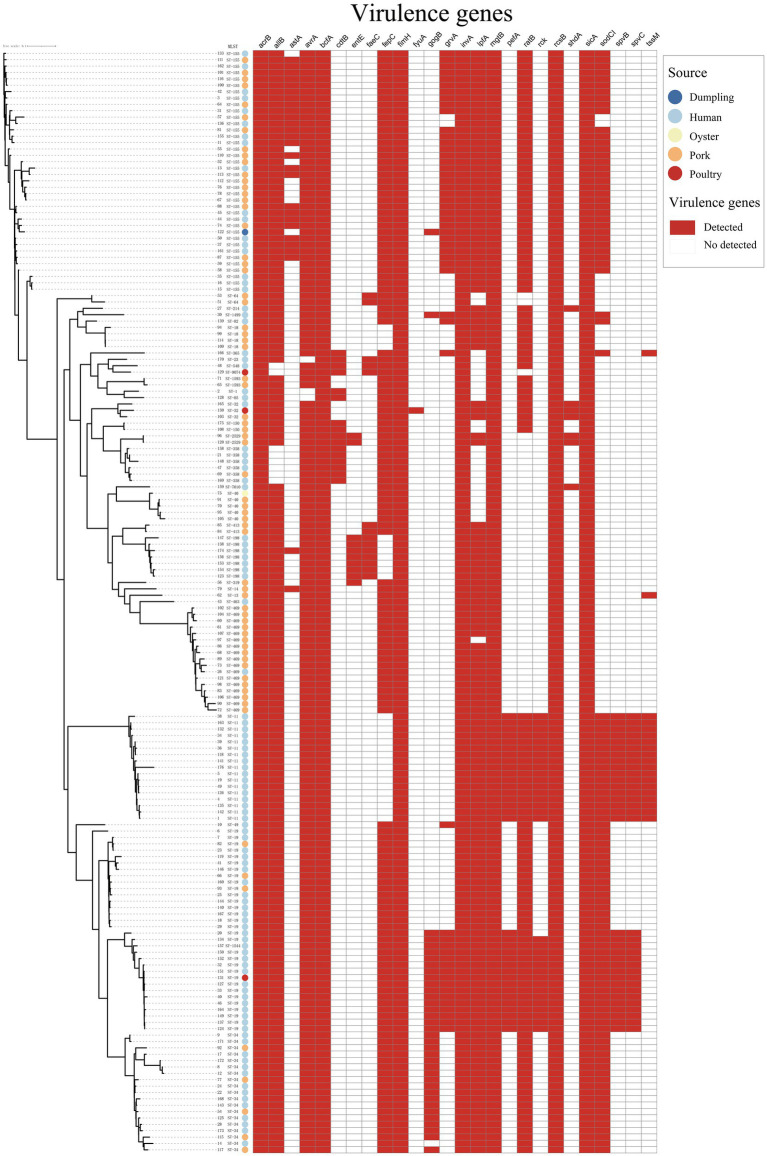
Presence/absence heatmap of virulence genes detected by WGS (*n* = 173). Rows correspond to isolates annotated by serotype and source (clinical vs. food); a core-genome phylogeny (left) clusters related isolates. Columns represent virulence genes ordered by functional category. Dark squares indicate presence; light squares indicate absence.

To further characterize virulence potential, we screened WGS data for known virulence-associated genes; results are summarized in Supplementary Table S2 and visualized as a presence/absence heatmap (Figure 2, Supplementary Figure S2). A total of 170 distinct virulence genes were detected across the collection; per-isolate counts ranged from 99 to 129, and 81 genes were ubiquitous among all isolates. These conserved loci are mainly related to adherence, effector delivery systems and nutritional/metabolic functions. Notably, the typhoid-toxin gene *cdtB* was detected not only in *S*. Typhi and *S*. Paratyphi A, but also in isolates of *S.* Oranienburg, *S.* Minnesota, *S.* Javiana, *S.* Weltevreden, *S.* Bovismorbificans (ST150) and all *S.* Goldcoast isolates. The antivirulence gene *grvA* was present in 53 isolates, predominantly in serovar *S.* London (*n* = 33) and *S*. Typhimurium (*n* = 16), and occurred in both human- and food-derived strains. Comparison among the five dominant serotypes revealed variation in 18 virulence genes. *S*. Typhimurium harbored 15 of these genes, *S*. Enteritidis 13, *S.* London and *S*. I 4,[5],12:i:- each 9, while *S.* Rissen carried only 2. Several adherence and effector-related genes (e.g., *lpfD*, *ratB*, *sodC1*, *sseI*, and *sspH2*) were present in the four serotypes above but absent in *S.* Rissen. Genes implicated in serum resistance and plasmid-associated virulence (*rck*, *pefABCD*, *spvB*, *spvC*) were observed in all *S*. Enteritidis and in a subset of *S*. Typhimurium. Overall, *S.* Rissen showed a virulence profile similar to *S.* Derby, with complementary presence/absence patterns for *lpf* and *sspH1* loci. These patterns suggest serotype-associated variability in virulence gene repertoires that may influence pathogenic potential.

### Phylogenetic and transmission analysis

**Figure 3:**
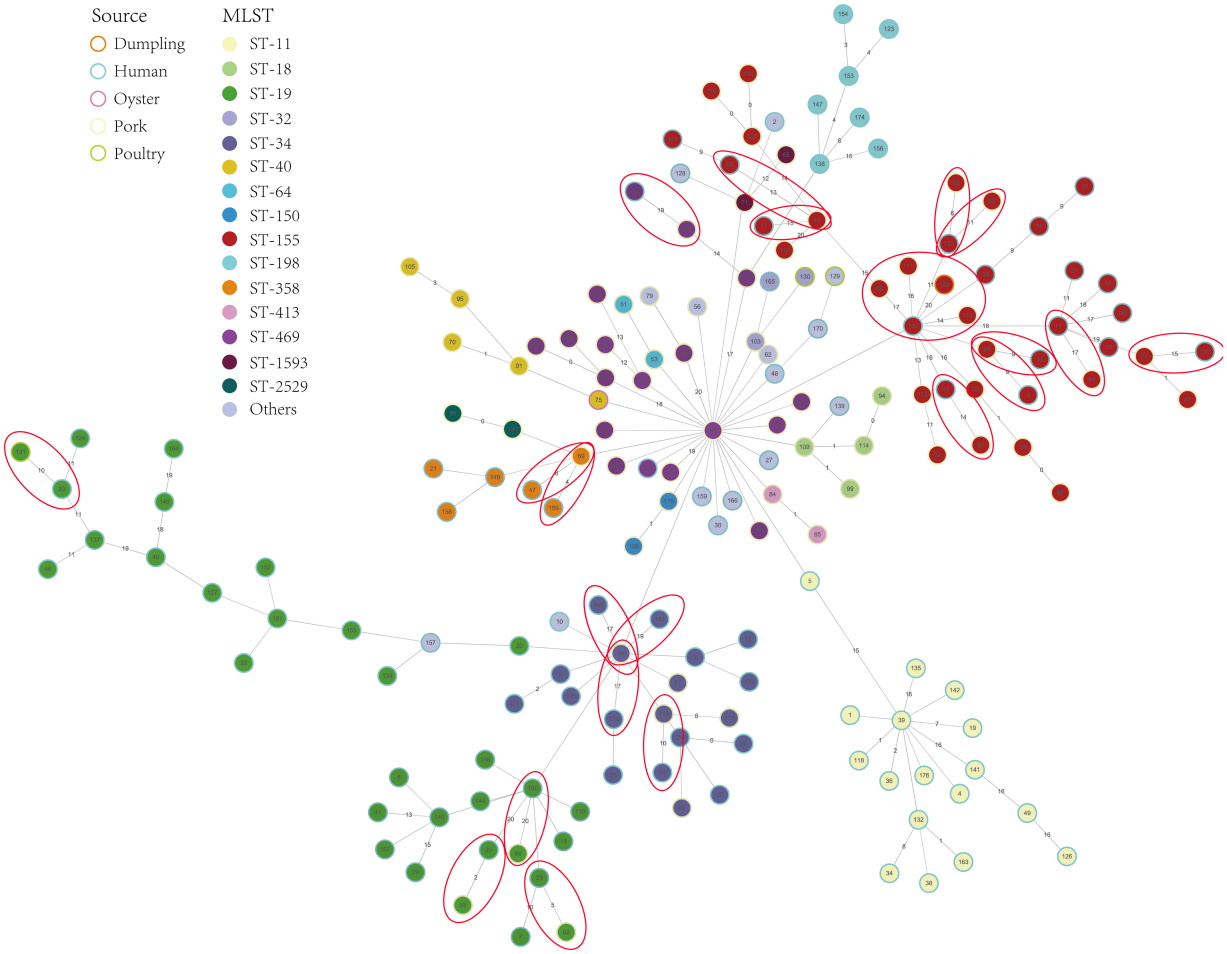
Minimum spanning tree based on core-genome SNPs. Node color denotes MLST, node outline denotes source. Examples of closely related food-human links (cgSNP ≤ 20) are circled in red.

To examine relatedness among the 173 *Salmonella* isolates, we combined a core-genome phylogeny with serovar-/ST-specific minimum spanning tree (MST) analyses based on core-genome SNPs which demonstrates clear clustering by serotype/ST: isolates of the same serovar generally group within discrete clades, reflecting strong phylogenetic coherence. Notable relationships include separation of *S*. Typhimurium into a major ST19 cluster and a related subgroup containing the *S*. I 4,[5],12:i:-; a single *S*. Typhimurium assigned to ST1544 groups within the broader ST19-associated clade. *S.* Bovismorbificans isolates of ST150 and ST1499 are phylogenetically distinct: ST150 clusters near *S.* Goldcoast, whereas ST1499 clusters with *S.* Litchfield, *S.* Muenchen and *S.* Manhattan. MST analyses (Figure 3, Supplementary Figure S3), colored by MLST and annotated by isolate source, were used to explore potential clonal transmission between food and human origins. Using a conservative core-genome SNP threshold of ≤20 to indicate close genetic relatedness (Mao et al., 2025), we observed multiple instances consistent with probable recent transmission or a common source: among *S*. Typhimurium/*S*. I 4,[5],12:i:-, five pork isolates were closely related to eight clinical isolates (pairwise SNPs 2–20); for *S.* London, food and clinical isolates co-clustered at six nodes with pairwise SNPs of 9–19; within *S.* Goldcoast, ST2529 formed a distinct sublineage separated from ST358, and one ST358 pork isolate was closely related to two clinical isolates (pairwise SNPs = 4 and 6, respectively). For *S.* Rissen, the sole clinical isolate clustered closely with one pork isolate (pairwise SNPs = 19). These patterns, observed across multiple serovars and STs, support occurrences of probable food-human transmission and local clonal spread in the study region.

## Discussion

*Salmonella* originating from animal reservoirs can be transmitted to humans through the food chain, causing gastroenteritis and occasionally invasive disease; retail meat is an important vehicle for human salmonellosis (Lyu et al., 2021). By integrating serovar distribution, antimicrobial resistance phenotypes, and WGS-based population structure for 173 isolates from retail foods (predominantly pork) and diarrheal patients in Ruian, our study suggests that local salmonellosis risk is shaped not only by the presence of particular serovars, but also by how specific lineages are maintained, amplified, and disseminated across the pork production and retail continuum. While our food isolates were predominantly derived from pork, this reflects the high prevalence of *Salmonella* in pork products within our study region. Therefore, our conclusions regarding the transmission pathways of *Salmonella* are specifically reflective of pork-associated risks, which may not be broadly generalizable to all food categories.

A notable epidemiological feature in Ruian is the shared predominance of *S.* London in both pork and clinical isolates. This differs from reports from other regions where *S.* Derby, *S.* Typhimurium, or *S.* I 4,[5],12:i:- frequently dominate pork-associated *Salmonella* (Wang et al., 2022; Zhang et al., 2018). Such a distinctive serovar profile is more consistent with region-specific transmission opportunities and selection pressures along the local pork chain than with random introductions alone. Persistence niches in farms, transport/lairage, slaughter and processing environments, and retail settings can repeatedly seed products and enable lineage expansion over time, and contamination can occur at multiple stages from pre-harvest to retail (Campos et al., 2019). In this context, the low genetic diversity observed for *S.* London (ST155) is compatible with local clonal expansion, whereas higher within-serovar diversity in other serovars implies more heterogeneous ecological niches and/or repeated introductions. These contrasting population structures have practical implications: persistent, low-diversity clones argue for identifying and disrupting reservoirs and cross-contamination points, while more diverse serovar populations argue for broader source attribution and risk mapping.

Phylogenomic evidence indicates possible linkage between pork and human infections. Core-genome SNP minimum spanning tree analyses identified multiple mixed-source clusters meeting a conservative close-relatedness threshold (≤20 SNPs) across serovars recovered from both sources (*S*. Typhimurium/*S.* I 4,[5],12:i:-, *S.* London, *S.* Goldcoast, and *S.* Rissen), suggesting potential recent transmission or shared sources linking pork and clinical cases. At the same time, the absence of food isolates for certain clinically important serovars (e.g., *S*. Enteritidis, *S.* Kentucky) and the low prevalence of *S*. Typhimurium in pork indicate that pork is unlikely to be the sole transmission route in Ruian, and that additional sources-other foods, water, environment, or animal contact-may also contribute. This dual signal supports strengthening pork-chain controls while expanding surveillance beyond a single commodity to avoid underestimating non-pork pathways.

The discrepancy observed for *S.* Rissen, which was common in pork but uncommon among clinical isolates, can be interpreted using a virulence–exposure framework. Exposure may be frequent, yet the probability of symptomatic illness and healthcare presentation may be lower. In our dataset, *S.* Rissen lacked several virulence-associated genes (e.g., *lpfD*, *ratB*, *sodC1*, *sseI*, *sspH2*) that were present in dominant clinical serovars such as *S*. Typhimurium, *S.* I 4,[5],12:i:-, *S*. Enteritidis, and *S.* London. Previous studies have reported its less virulence than *S*. Typhimurium in mouse models and high rate (about 50%) of asymptomatic human infection (Elbediwi et al., 2021; Xu X. et al., 2020; Zhou et al., 2020). The distinct difference in isolation rates of *S*. Rissen between pork and clinical sources, its comparatively reduced virulence-gene repertoire, as well as the high proportion of asymptomatic infections, collectively suggest a lower virulence potential of this serovar. These findings emphasize that prevalence in food does not necessarily translate proportionally into clinical burden, and that integrating virulence profiling with epidemiology may improve prioritization of serovars for intervention.

From a clinical and stewardship perspective, the high resistance rates to commonly used agents in clinical isolates (e.g., amoxicillin, piperacillin, and minocycline) underscore sustained selective pressure and reinforce the need for rational antimicrobial use. The significant differences in AMR profiles between *S*. Typhimurium and *S.* London suggest that profiles are partly lineage-associated and shaped by the interplay of antimicrobial use, mobile genetic elements, and clonal expansion (Liu et al., 2021). Furthermore, our findings highlight the importance of continuous surveillance of AMR profiles, as they can inform treatment guidelines and public health strategies. The observation that only 30.19% of isolates were resistant to ciprofloxacin and 22.64% to levofloxacin is notable, particularly given the rising trend of fluoroquinolone resistance globally. The lack of fluoroquinolone-related genes in resistant strains raises questions about alternative resistance mechanisms, such as efflux pumps or mutations in target genes, which may not be detected by standard gene screening methods. Additionally, the significant discordance between genotypic and phenotypic resistance profiles, as seen with tetracycline resistance genes, where the detection rate was only 25.29% compared to a phenotypic resistance rate of 42.20% for minocycline, indicates that WGS-based AMR inference should complement, rather than replace, phenotypic susceptibility testing (Piddock, 2016; Tang et al., 2022). This highlights the necessity for a multifaceted approach to understanding AMR, as discrepancies may arise from regulatory variations, efflux/permeability changes, or uncharacterized target alterations that are not captured by routine genetic screening. Overall, these data support a One Health prevention strategy for Ruian that couples WGS-enabled surveillance with interventions across the pork chain, focusing on interrupting persistence and cross-contamination, while also broadening monitoring to other plausible sources of infection. Downstream, reinforcing safe food handling and kitchen hygiene remains essential to reduce consumer-level exposure.

## Limitations

There were several limitations in this study. Firstly, food sampling was largely limited to retail pork, and other potentially important sources (e.g., other meats, eggs, produce, water, environment, companion animals) were not systematically included. This constrained our ability to estimate the relative contribution of pork versus non-pork pathways for serovars detected in humans but not recovered from pork. Secondly, the study relied on archived isolates and may have had uneven sampling intensity across years and sites, which could bias apparent temporal patterns and may not fully capture short-term fluctuations in circulating serovars and clones. Thirdly, epidemiological metadata were limited, including detailed patient exposure histories and fine-scale traceability information for retail products. This restricted formal source attribution and limited the ability to link genomic clusters to specific contamination points or behavioral risk factors.

## Data Availability

The datasets presented in this study can be found in online repositories. The names of the repository/repositories and accession number(s) can be found at: https://www.ncbi.nlm.nih.gov/, PRJNA1436825.

## Footnotes

1
https://pubmlst.org/bigsdb?db=pubmlst_salmonella_seqdef

## References

[ref1] Baek S.-H. Lim C.-G. Park J.-I. Seo Y.-B. Nam I.-S. (2024). Investigation of *Salmonella enteritidis* growth under varying temperature conditions in liquid whole egg: proposals for smart Management Technology for Safe Refrigerated Storage. Foods 13:3106. doi: 10.3390/foods13193106, 39410142 PMC11475776

[ref2] Barza M. (2002). Potential mechanisms of increased disease in humans from antimicrobial resistance in food animals. Clin. Infect. Dis. 34:S123–S125. doi: 10.1086/340249, 11988882

[ref3] Besser J. M. (2018). *Salmonella* epidemiology: a whirlwind of change. Food Microbiol. 71, 55–59. doi: 10.1016/j.fm.2017.08.018, 29366469

[ref4] Campos J. Mourão J. Peixe L. Antunes P. (2019). Non-typhoidal *Salmonella* in the pig production chain: a comprehensive analysis of its impact on human health. Pathogens 8:19. doi: 10.3390/pathogens8010019, 30700039 PMC6470815

[ref5] Chen J. Ed-Dra A. Zhou H. Wu B. Zhang Y. Yue M. (2022). Antimicrobial resistance and genomic investigation of non-typhoidal *Salmonella* isolated from outpatients in Shaoxing city, China. Front. Public Health 10:988317. doi: 10.3389/fpubh.2022.988317, 36176509 PMC9513250

[ref6] Cheng R. A. Eade C. R. Wiedmann M. (2019). Embracing diversity: differences in virulence mechanisms, disease severity, and host adaptations contribute to the success of nontyphoidal *Salmonella* as a foodborne pathogen. Front. Microbiol. 10:1368. doi: 10.3389/fmicb.2019.01368, 31316476 PMC6611429

[ref7] CLSI (Clinical and Laboratory Standards Institute). (2024). Performance Standards for Antimicrobial Susceptibility Testing. Wayne: Clinical and Laboratory Standards Institute (CLSI). Available online at: https://clsi.org/shop/standards/m100/ (Accessed September 15, 2024).

[ref8] Croucher N. J. Page A. J. Connor T. R. Delaney A. J. Keane J. A. Bentley S. D. . (2015). Rapid phylogenetic analysis of large samples of recombinant bacterial whole genome sequences using Gubbins. Nucleic Acids Res. 43:e15. doi: 10.1093/nar/gku1196, 25414349 PMC4330336

[ref9] Deng X. Li S. Xu T. Zhou Z. Moore M. M. Timme R. . (2025). *Salmonella* serotypes in the genomic era: simplified *Salmonella* serotype interpretation from DNA sequence data. Appl. Environ. Microbiol. 91:e0260024. doi: 10.1128/aem.02600-24, 39992117 PMC11921320

[ref10] Dong Q. L. Barker G. C. Gorris L. G. M. Tian M. S. Song X. Y. Malakar P. K. (2015). Status and future of quantitative microbiological risk assessment in China. Trends Food Sci. Technol. 42, 70–80. doi: 10.1016/j.tifs.2014.12.003, 26089594 PMC4460287

[ref11] Dos Santos A. M. P. Ferrari R. G. Conte-Junior C. A. (2019). Virulence factors in *Salmonella Typhimurium*: the sagacity of a bacterium. Curr. Microbiol. 76, 762–773. doi: 10.1007/s00284-018-1510-4, 29785632

[ref12] Ehuwa O. Jaiswal A. K. Jaiswal S. (2021). *Salmonella*, food safety and food handling practices. Foods 10:907. doi: 10.3390/foods10050907, 33919142 PMC8143179

[ref13] Elbediwi M. Shi D. Biswas S. Xu X. Yue M. (2021). Changing patterns of *Salmonella enterica* Serovar Rissen from humans, food animals, and animal-derived foods in China, 1995–2019. Front. Microbiol. 12:702909. doi: 10.3389/fmicb.2021.702909, 34394048 PMC8358327

[ref14] Fang L. Lin G. Li Y. Lin Q. Lou H. Lin M. . (2022). Genomic characterization of *Salmonella enterica* serovar Kentucky and London recovered from food and human salmonellosis in Zhejiang Province, China (2016–2021). Front. Microbiol. 13:961739. doi: 10.3389/fmicb.2022.961739, 36060737 PMC9437622

[ref15] Grimont P. A. D. Weill F. -X. (2007). Antigenic Formulae of the *Salmonella* Serovars. 9th Edn. Paris: Institut Pasteur.

[ref16] Lai J. Wu C. Wu C. Qi J. Wang Y. Wang H. . (2014). Serotype distribution and antibiotic resistance of *Salmonella* in food-producing animals in Shandong province of China, 2009 and 2012. Int. J. Food Microbiol. 180, 30–38. doi: 10.1016/j.ijfoodmicro.2014.03.030, 24786550

[ref17] Lamichhane B. Mawad A. M. M. Saleh M. Kelley W. G. Harrington P. J. Lovestad C. W. . (2024). Salmonellosis: an overview of epidemiology, pathogenesis, and innovative approaches to mitigate the antimicrobial resistant infections. Antibiotics 13:76. doi: 10.3390/antibiotics13010076, 38247636 PMC10812683

[ref18] Liu Y. Jiang J. Ed-Dra A. Li X. Peng X. Xia L. . (2021). Prevalence and genomic investigation of *Salmonella* isolates recovered from animal food-chain in Xinjiang, China. Food Res. Int. 142:110198. doi: 10.1016/j.foodres.2021.110198, 33773671

[ref19] Lyu N. Feng Y. Pan Y. Huang H. Liu Y. Xue C. . (2021). Genomic characterization of *Salmonella enterica* isolates from retail meat in Beijing, China. Front. Microbiol. 12:636332. doi: 10.3389/fmicb.2021.636332, 33897640 PMC8058101

[ref20] Magiorakos A.-P. Srinivasan A. Carey R. B. Carmeli Y. Falagas M. E. Giske C. G. . (2012). Multidrug-resistant, extensively drug-resistant and pandrug-resistant bacteria: an international expert proposal for interim standard definitions for acquired resistance. Clin. Microbiol. Infect. 18, 268–281. doi: 10.1111/j.1469-0691.2011.03570.x, 21793988

[ref21] Mao W. Jin F. Wang Y. Zhao J. Xu P. Yu Y. . (2025). Genomic characterization and global relatedness of multidrug-resistant *Salmonella* Goldcoast ST2529. BMC Genomics 26:708. doi: 10.1186/s12864-025-11903-4, 40745636 PMC12312370

[ref22] Piddock L. J. V. (2016). Assess drug-resistance phenotypes, not just genotypes. Nat. Microbiol. 1:16120. doi: 10.1038/nmicrobiol.2016.120, 27573119

[ref23] Soliani L. Rugna G. Prosperi A. Chiapponi C. Luppi A. (2023). *Salmonella* infection in pigs: disease, prevalence, and a link between swine and human health. Pathogens 12:1267. doi: 10.3390/pathogens12101267, 37887782 PMC10610219

[ref24] Tang B. Elbediwi M. Nambiar R. B. Yang H. Lin J. Yue M. (2022). Genomic characterization of antimicrobial-resistant *Salmonella enterica* in duck, chicken, and pig farms and retail markets in eastern China. Microbiol. Spectrum 10:e01257-22. doi: 10.1128/spectrum.01257-22, 36047803 PMC9603869

[ref25] Wang Z. Zhang J. Liu S. Zhang Y. Chen C. Xu M. . (2022). Prevalence, antimicrobial resistance, and genotype diversity of *Salmonella* isolates recovered from retail meat in Hebei Province, China. Int. J. Food Microbiol. 364:109515. doi: 10.1016/j.ijfoodmicro.2021.109515, 35030440

[ref26] Wu S. Hulme J. P. (2021). Recent advances in the detection of antibiotic and multi-drug resistant *Salmonella*: an update. Int J Mol Sci 22:3499. doi: 10.3390/ijms22073499, 33800682 PMC8037659

[ref27] Xu X. Biswas S. Gu G. Elbediwi M. Li Y. Yue M. (2020). Characterization of multidrug resistance patterns of emerging *Salmonella enterica* Serovar Rissen along the food chain in China. Antibiotics 9:660. doi: 10.3390/antibiotics9100660, 33007986 PMC7600917

[ref28] Xu Y. Zhou X. Jiang Z. Qi Y. Ed-dra A. Yue M. (2020). Epidemiological investigation and antimicrobial resistance profiles of *Salmonella* isolated from breeder chicken hatcheries in Henan, China. Front. Cell. Infect. Microbiol. 10:497. doi: 10.3389/fcimb.2020.00497, 33042870 PMC7522330

[ref29] Yan S. Jiang Z. Zhang W. Liu Z. Dong X. Li D. . (2023). Genomes-based MLST, cgMLST, wgMLST and SNP analysis of *Salmonella Typhimurium* from animals and humans. Comp. Immunol. Microbiol. Infect. Dis. 96:101973. doi: 10.1016/j.cimid.2023.101973, 36989679

[ref30] Yang X. Wu Q. Zhang J. Huang J. Chen L. Wu S. . (2019). Prevalence, bacterial load, and antimicrobial resistance of *Salmonella* serovars isolated from retail meat and meat products in China. Front. Microbiol. 10:2121. doi: 10.3389/fmicb.2019.02121, 31608021 PMC6771270

[ref31] Yoshida C. E. Kruczkiewicz P. Laing C. R. Lingohr E. J. Gannon V. P. J. Nash J. H. E. . (2016). The *Salmonella* In Silico Typing Resource (SISTR): an open web-accessible tool for rapidly typing and subtyping draft *Salmonella* genome assemblies. PLoS One 11:e0147101. doi: 10.1371/journal.pone.0147101, 26800248 PMC4723315

[ref32] Zhang L. Fu Y. Xiong Z. Ma Y. Wei Y. Qu X. . (2018). Highly prevalent multidrug-resistant *Salmonella* from chicken and pork meat at retail markets in Guangdong, China. Front. Microbiol. 9:2104. doi: 10.3389/fmicb.2018.02104, 30258419 PMC6143800

[ref33] Zhou A. Li J. Xu Z. Ni J. Guo J. Yao Y.-F. . (2020). Whole-genome comparative and pathogenicity analysis of *Salmonella enterica* subsp. *enterica* Serovar Rissen. G3 (Bethesda) 10, 2159–2170. doi: 10.1534/g3.120.401201, 32358017 PMC7341144

