# Supplementary figures and images for: Genomic characterization of Salmonella isolates from food and diarrheal patients in Ruian, China
Source: Front Microbiol. 2026 Apr 29;17:1840296. doi: 10.3389/fmicb.2026.1840296 (PMC13168153; doi:10.3389/fmicb.2026.1840296)

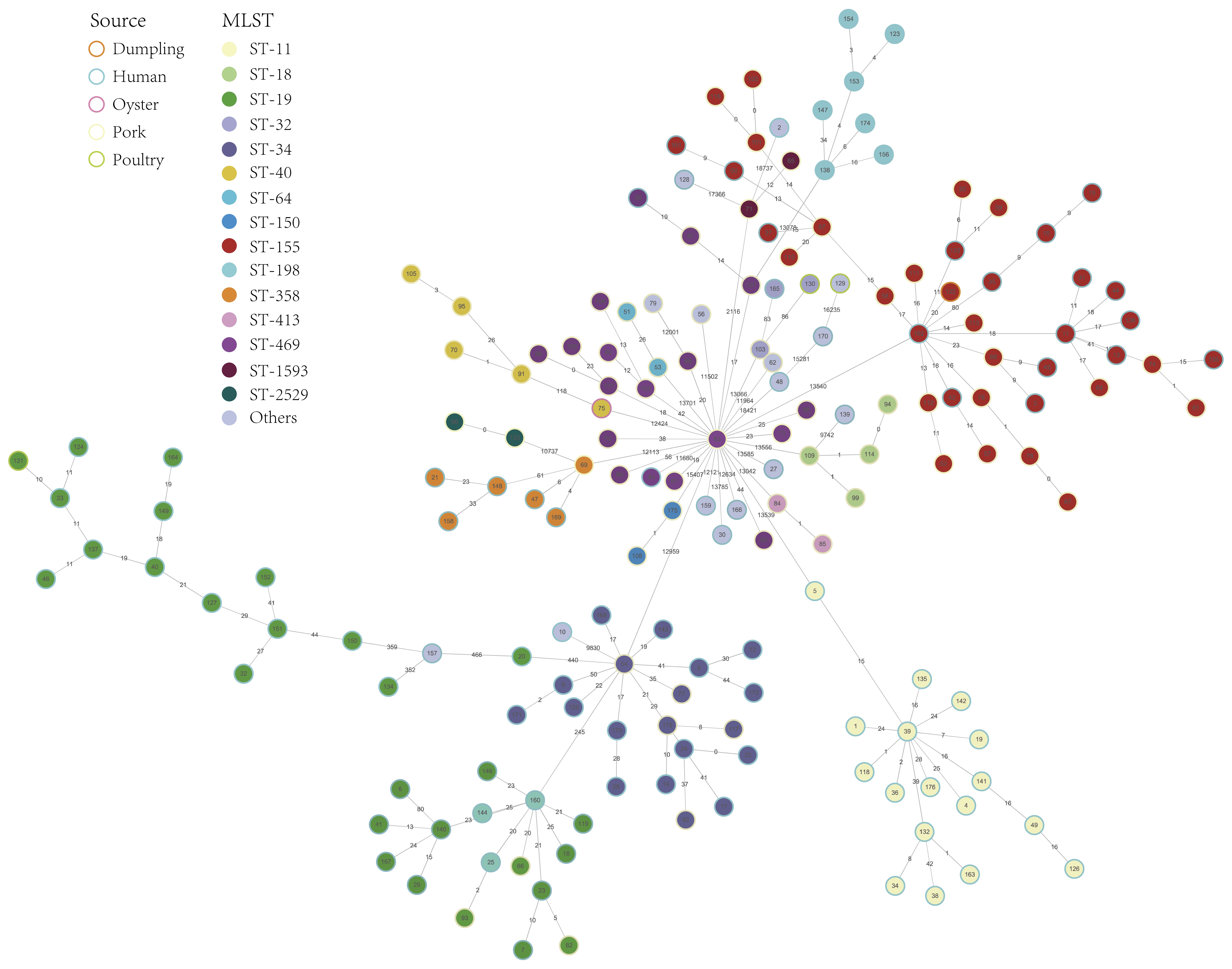

Supplement: Supplementary file 5 [file Image_3.JPEG]
